# Neutrophils in tissue injury and repair

**DOI:** 10.1007/s00441-017-2785-7

**Published:** 2018-01-30

**Authors:** Jing Wang

**Affiliations:** Division of Inflammation Biology, Institute of Enzyme Research, Tokushima University, Tokushima, Japan

**Keywords:** Neutrophils, Injury, Repair, Regeneration, Angiogenesis

## Abstract

As one of the first defenders of innate immune response, neutrophils make a rapid and robust response against infection or harmful agents. While traditionally regarded as suicidal killers that cause collateral tissue damage, recent findings on neutrophil extracellular trap formation, heterogeneity and plasticity and novel reparative functions have expanded our understanding of their diverse roles in health and disease. This review summarizes our current understanding of neutrophil-associated tissue injury, highlighting the emerging roles of neutrophil extracellular traps. This review will also focus on scrutinizing the roles of neutrophils in tissue repair and regeneration and will examine data on unexpected aspects of involvement of neutrophils in regulating normal tissue homeostasis.

## Introduction

Neutrophils are the predominant immune cell in human blood, where they patrol and protect the host from pathogens and other harmful reagents (Kolaczkowska and Kubes 2013). During infection, neutrophils are mostly viewed as playing a beneficial role to the host, as neutropenic patients are at high risk for mortality from infection; although these robust effector functions may also lead to tissue damage. In cases where the inflammatory process is generated by injury itself, which is also known as sterile inflammation, it becomes more controversial as to whether neutrophils themselves have any beneficial effects that may contribute to repair the parenchyma or vasculature. The outcome of the neutrophil response will most likely to be context-dependent, which includes but is not limited to, the trigger of the inflammatory response, the tissue environment and other cell types that interact with neutrophils. Altogether, these factors collectively determine whether an inflammatory response is a positive feedback amplification progress or a negative feedback self-limiting progress. Importantly, it is still largely unknown how these progresses are determined and the key mediators that trigger the conversion from physiological tissue repair and regeneration to pathological tissue damage and chronic diseases remains to be defined.

Novel technologies, such as intravital microscopy and transgenic animals, have helped tremendously to expand our understanding of neutrophil homeostasis and effector functions. Development of new microscopes that allow visualization of deep tissue and fast-moving cells provides promising experimental tools to study neutrophil functions in vivo. So far, studies using intravital imaging have made a huge contribution to our understanding of the neutrophil recruitment paradigm in different tissue environments and in various inflammatory conditions (Kolaczkowska and Kubes 2013; Nourshargh and Alon 2014). However, it has been only recently that researchers have started moving forward, looking at neutrophil dynamics and events after their recruitment to the tissue. It has been difficult to specifically label neutrophils residing in interstitial tissue with fluorescent tags and the widely used LysM reporter strain has difficulties distinguishing between neutrophils and monocytes/macrophages, especially in inflamed tissue, where these cells are present in large numbers. Recently, a new strain of mouse using the more specific promoter Ly6G, which is only expressed in neutrophils among the hematopoietic compartment, has been established (Hasenberg et al. 2015). This strain therefore allows for the specific and unequivocal investigation of neutrophil functions in vivo (Zec et al. 2016; Wang et al. 2017). These developments have thus spawned more complex studies regarding the role of neutrophils in the context of homeostatic progress, such as tissue repair and regeneration.

In this review, I will briefly discuss studies linking neutrophils to collateral tissue damage, especially the emerging roles of neutrophil extracellular traps. I will focus on the roles of neutrophils in the context of tissue repair and regeneration, and discuss several different strategies that are employed by neutrophils that contribute to the restoration of homeostasis. I will review the role of the neutrophils in several conditions in which evidence has accumulated that indicates their contributions to repair, highlighting the need for further understanding of neutrophil biology for the development of proper therapeutic targets.

## Neutrophil recruitment to tissue injury

Neutrophils are developed in the bone marrow from hematopoietic stem cells in a process called “granulopoiesis”. After being released into blood, neutrophils patrol the circulation until they encounter inflammatory signals. The first signals that are responsible for early neutrophil recruitment are released from damaged and necrotic cells after tissue injury and are likely to be damage-associated molecular patterns (DAMPs) (Pittman and Kubes 2013). These DAMP molecules include DNA, histones, high mobility group protein B1 (HMGB1), N-formyl peptides, Adenosine triphosphate (ATP), interleukin-1α (IL-1α) and many others (Chen and Nunez 2010). Many DAMPs can act as chemoattractants and are sensed by neutrophils often through G-protein-coupled receptors (GPCRs). Alternatively, DAMPs released from damaged cells can activate surrounding tissues and induce the production of chemokines and lipid mediators, for example C-X-C motif chemokine ligand 8 (CXCL8) and leukotriene B_4_ (LTB_4_) (Heijink et al. 2015; Lammermann et al. 2013), both are strong inducers of neutrophil chemotaxis. Once released by both immune cells (neutrophils, macrophages and T cells) and non-hematopoietic cells (epithelial and endothelial cells) in response to injury and infection, CXCL8 can bind to glycosaminoglycans on cell walls and in the extracellular matrix to create chemokine gradients along the tissues and structures through which neutrophils migrate (Webb et al. 1993). A study transplanted fluorescently tagged CXCL8-expressing cells into zebrafish larvae and then observed CXCL8 accumulated locally around the transplanted cells but which then spread outwards into the vasculature, forming immobilized gradients (Sarris et al. 2012). In mouse models of acute hepatic injury, such intravascular extracellular matrix-bound chemokine gradients have also been observed reaching as far away as 650 μm (McDonald et al. 2010).

DAMPs that are released from damaged cells activate the production of these chemokines and lipid mediators through several different mechanisms. For example, DAMPs can activate endothelial cells to release the pre-made stores of chemokines via exocytosis. DAMPs can also activate transcriptional pathways, such as nuclear factor-κB (NF-κB), which results in the production of chemokines and lipid mediators. ATP-mediated activation of the NLRP3 inflammasome has been associated with persistent neutrophil recruitment, presumably through the release of IL-1 molecules (Rider et al. 2011). IL-1α has recently been described as a major DAMP molecule involved in the initiation of sterile inflammation and has been shown to be important for the recruitment of neutrophils. IL-1α released by necrotic cells was crucial for the production of CXCL1, which also recruits neutrophils (Chen and Nunez 2010).

It is worth mentioning that necrotic cells that are present in tissue injury probably release multiple types of DAMP molecules and how these molecules orchestrate together to recruit neutrophils remains to be studied. Nevertheless, neutrophil recruitment is usually dramatically amplified through several positive feedback mechanisms (Nemeth and Mocsai 2016).

## Heterogeneity and plasticity of neutrophils: phenotypes and functions

Heterogeneity of neutrophils has been defined at several levels: (1) nuclear appearance (band cells, mature and hypersegmented neutrophils); (2) density; (3) surface receptor expression profiles associated with distinct neutrophil subsets. During the last decade, evidence has been accumulating for the existence of neutrophil subsets in different models, which are briefly summarized in Table 1. Some of these subsets appear to be disease- or tissue-specific, while the factors that govern the generation of the heterogeneity are largely uninvestigated (Silvestre-Roig et al. 2016). A few of these subsets have been identified based on their injury or repair functions. Emerging technologies such as mass cytomery and single-cell sequencing have greatly improved our understanding of the heterogeneity of immune systems and have contributed to identify novel, distinct immune cell subsets and further application of these novel technologies may lead to a better understanding of the heterogeneity of neutrophil populations.

**Table 1:** Neutrophil subsets in homeostatic and pathological conditions; in contrast to other immune cell populations, the idea of neutrophil heterogeneity has received less attention, hence I have listed several emerging discoveries that have suggested phenotypic and functional heterogeneity of neutrophils

Subset markers	Species	Expression pattern	Contribution to tissue injury/repair	Reference
Olfactomedin 4^+^ neutrophils	Human	Healthy; inflammatory arthritis	Associated with NET-releasing	(Welin et al. 2013)
CD177^+^ Proteinase 3^+^neutrophils	Human	Increased in SLE, ANCA-associated systemic vasculitis	CD177 antoantibody	(Bauer et al. 2007)
CD16^hi^CD62L^dim^	Human	10–15% in human endotoxemia	Immunosuprression	(Pillay et al. 2012; Tak et al. 2017)
CXCR4^+^	Human Mouse	Healthy	Aged neutrophils	(Hartl et al. 2008)
CD49^+^	Human Mouse	Increased in atopic diseases (human) ~50% in Sendai virus infection (mouse)	Associated with allergic diseases	(Sigua et al. 2014)
ICAM-1^+^CD54^+^	Human Mouse	Ischemia/reperfusion	Distal organ injury	(Woodfin et al. 2011; Wu et al. 2016)
N1/N2	Mouse	Cancer Myocardia infarction	N2 neutrophils may contribute to repair	(Fridlender et al. 2009; Ma et al. 2016)
CD63^+^ MHCII^+^CD80^+^CD294^+^	Human	Cystic fibrosis	Suppressing T cell function	(Ingersoll et al. 2015; Tirouvanziam et al. 2008)
MMP-9^+^	Human Mouse	Healthy Transplanted islet	Promote revascularization	(Christoffersson et al. 2010)
TCRαβ-expressing neutrophils	Human Mouse	Healthy	Unknown	(Puellmann et al. 2006)
LDGs	Human	Healthy SLE patients	Induce vascular damage	(Denny et al. 2010)

Although it might be an oversimplified concept, in general, macrophages have been described as having pro-inflammatory M1 and anti-inflammatory M2 phenotypes (Gordon and Taylor 2005). Similar to this concept, different functionally distinct neutrophil subsets were first observed in cancer, where pro-inflammatory, anti-tumoral (N1) and anti-inflammatory pro-tumoral (N2) phenotypes have been found (Fridlender et al. 2009). A recent study demonstrated temporal neutrophil polarization following myocardial infarction (MI) (Ma et al. 2016). Data from the same study also suggested that, similar to macrophages, in vitro, peripheral blood neutrophils can be polarized to pro-inflammatory N1 by lipopolysaccharide (LPS) and interferon-γ or anti-inflammatory N2 by interleukin-4 (IL-4). In vivo, cells with the N1 phenotype are the predominant neutrophils in the heart early on following MI, whereas the N2 subset increased over time, supporting its role in the resolution of inflammation and tissue repair. Although further work is needed to clearly demonstrate the functions and phenotypic profiles of this N2 subset, it will be interesting to examine whether such neutrophil polarization will also occur in the context of other inflammatory diseases. Another intriguing question arising from this study is whether the N1 and N2 subsets are plastic and cells can be stimulated to change from one subset to the other. Monocyte/macrophage subsets conversion has been intensively investigated and their capability to switch phenotypes provides great therapeutic potentials to modify these cells (Kratofil et al. 2017). However, neutrophils have a much shorter life-span and this reprogramming may not be as easy to induce. Nevertheless, programming neutrophils to the anti-inflammatory N2 subtype by a Peroxisome proliferator-activated receptor gamma PPARγ agonist has been used to obtain a beneficial outcome in stroke (Cuartero et al. 2013).

## Neutrophil associated tissue injury

Neutrophils contribute to tissue injury by amplifying the inflammatory response and direct release of toxic effectors. The effectors from neutrophils that may contribute to tissue damage have been discussed in other reviews and will not be discussed in detail here (Kruger et al. 2015; Segel et al. 2011). In general, reactive oxygen species (ROS) such as superoxide and hydrogen peroxide and non-oxidative mechanisms such as those involving proteolytic enzymes and antimicrobial proteins are considered to be able to cause tissue damage (Wilgus et al. 2013). However, those effectors do not always damage the tissue. For example, it has been shown that neither neutrophils nor ROS appear to be the causative agent of tissue damage during *Pneumocystic* infection (Swain et al. 2004). Some of these factors may even have a beneficial contribution in certain inflammatory conditions. A recent study suggested that myeloperoxidase (MPO), released by neutrophils, diminishes the toxic effects and protects the host from LPS-induced fatal tissue injury (Gaut et al. 2001; Reber et al. 2017). It has been proposed that MPO may regulate the acute inflammatory responses by modulating the formation of lipid mediators (Kubala et al. 2010). MPO is also responsible for generating toxic ROS and it remains under investigation how the enzymic activity of MPO is regulated in inflammatory responses that have different outcomes.

## Neutrophil extracellular trap-induced tissue damage

The concept of extracellular killing by neutrophils using neutrophil extracellular traps (NETs) has received much attention during the past decade (Brinkmann et al. 2004). Recent evidence has emerged suggesting that NETs and their components, can be injurious to host tissue (Clark et al. 2007) and thereafter contribute to the development of many noninfectious diseases, such as lung injury, systemic lupus erythematous (SLE), rheumatoid arthritis (RA), diabetes, atherosclerosis, thrombosis and cancer (Jorch and Kubes 2017). NETs are released when certain intracellular pathways are activated in neutrophils. Often, during formation of NETs, neutrophils die and this process is generally referred to as NETosis (Steinberg and Grinstein 2007). NETs contain nuclear contents as well as granular and cytosolic proteins, which result in the potential presentation of auto-antigens to the host immune system and the release of DAMPs that could further amplify ongoing immune reactions and cause tissue injury. Several strategies have been proposed to interfere with NETs, including digestion of NET-DNA with DNase or targeting NET-associated proteins. These approaches have demonstrated that blocking NET formation results in less tissue damage (Kolaczkowska et al. 2015). Clinically, DNase has been successfully used to treat cystic fibrosis patients and the beneficial effect may be due to the digestion of NETs (Manzenreiter et al. 2012; Papayannopoulos et al. 2011). However, in monosodium urate crystal-induced acute inflammation (gout), aggregated NETs promote the resolution of neutrophilic inflammation by degrading cytokines and chemokines via serine proteases and disrupting neutrophil recruitment and activation (Schauer et al. 2014). Whether NETs may have a beneficial role in other inflammatory conditions needs to be studied in more detail.

## Contribution of neutrophils to tissue repair

**Fig. 1:**
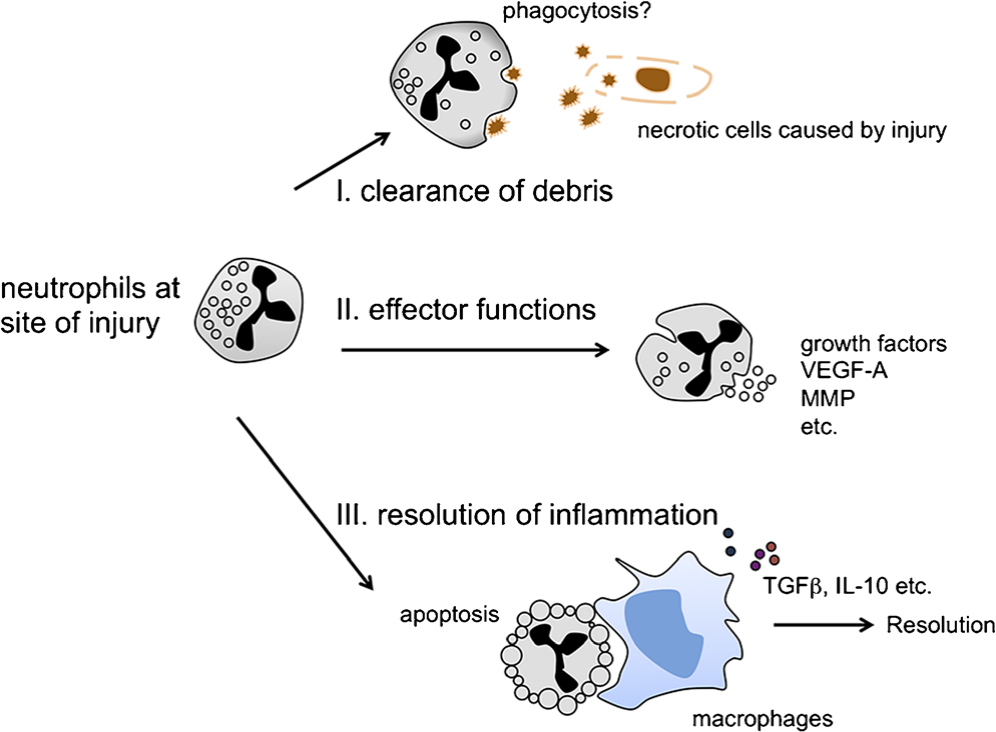
Neutrophil-mediated repair response. Three possible strategies that are adopted by neutrophils to promote tissue repair. *I* Neutrophils can clear necrotic cellular debris. A detailed mechanism in this progress remains to be studied. *II* Neutrophils release effectors that promote angiogenesis and regeneration; only “beneficial” effectors are listed in the figure. *III* Phagocytosis of apoptotic neutrophils results in release of anti-inflammatory and reparative cytokines

The inflammatory response after tissue injury is a dynamic process composed of sequential steps and aimed at restoring tissue architecture and function. Depending on the type of tissue where injury occurs, there are three possible strategies that may be adopted by neutrophils to repair a damaged tissue (Fig. 1). First, as professional phagocytes, neutrophils can remove tissue debris at the site of injury. The debris disposal mechanisms seem to be very effective, as cellular remnants are usually rare in physiological conditions. However, the identity of cellular debris and how the neutrophils recognize and dispose of them are under current extensive investigations. Second, mature neutrophils have more than 700 proteins including growth factors or pro-angiogenic factors stored in their segmented nucleus and granules (Dalli et al. 2013). Many can be rapidly released upon activation independent of transcription and thus directly contribute to regeneration and revascularization. And third, the most widely studied mechanism of neutrophil contribution to tissue repair is that neutrophils become apoptotic and are cleared by macrophages (Soehnlein and Lindbom 2010). This clearance process initiates a feed-forward pro-resolution programme that is characterized by the release of the tissue-repairing cytokines transforming growth factor-β (TGFβ) and interleukin-10 (IL-10). Thus, drugs that promote neutrophil apoptosis have a therapeutic potential to accelerate tissue repair (Robertson et al. 2014).

In the following sections, I will discuss the role of neutrophils in tissue repair in detail, citing literature that supports their important reparative functions. It is noteworthy that, although inflammation presumably evolved as an adaptive response for restoring homeostasis, many experimental models usually represent over-activated or inappropriate inflammatory responses, which fail to restore tissue homeostasis. Therefore, the contribution of neutrophils to tissue repair may not be properly evaluated in these models. It is possible that, even in these pathological models, neutrophils may still display some beneficial effects and the unbalance between beneficial and detrimental activities could favor the development of disease.

### Role of neutrophils in angiogenesis

Revascularization is part of the repair process following tissue injury. Newly formed blood vessels participate in provisional granulation tissue formation and provide nutrition and oxygen to growing tissues (Li et al. 2003). Angiogenesis is a dynamic process that is highly regulated by signals from both serum and the surrounding extracellular matrix (ECM) environment. Vascular endothelial growth factor (VEGF), angiopoietin, fibroblast growth factor (FGF) and TGFβ are among the most potent angiogenic cytokines. VEGF is a key player in blood vessel formation and has a direct chemotactic effect on endothelial cells. Both human and murine neutrophils have been demonstrated to be a source of VEGF (Gaudry et al. 1997; Gong and Koh 2010). In a corneal injury model, antibody-mediated neutrophil depletion severely inhibited corneal angiogenesis: a 90% reduction in new blood vessel length and neovascularized area compared with the control mice at day 5 after injury (Gong and Koh 2010). Interestingly, immunohistochemistry examination suggested that both corneal epithelial cells and infiltrating neutrophils express VEGF but no VEGF signal can be detected in the epithelial cells after neutrophil depletion. This suggested that infiltrating neutrophils not only produce VEGF themselves but could also interact with epithelial cells to induce the production of VEGF in the epithelial cells. Another study showed that isolated pancreatic islets transplanted into muscle did not revascularize in neutropenic mice, whereas intra-islet vasculature was restored after transplantation into wild-type mice, showing that recruited neutrophils are important in the initiation of angiogenesis (Christoffersson et al. 2010). Neutrophils are also an important storage site of another proangiogenic factor, matrix metalloproteinases (MMPs). MMPs are a family of zinc-dependent endopeptidases that are responsible for the degradation of extracellular matrix components and the release of VEGF and other growth factors bound to the extracellular matrix. Neutrophils are the only cells in the body that can release MMP-9 free of its endogenous inhibitor, tissue inhibitor of metalloproteinase and are therefore capable of delivering highly active MMP-9 to angiogenic sites (Ardi et al. 2007). Other angiogenic factors released from neutrophil granules that directly activate endothelial growth include the cathelicidin antimicrobial peptide, LL-37/hCAP-18 (Koczulla et al. 2003).

### Neutrophils in cutaneous wound healing

The most widely used tissue injury and repair model is probably the cutaneous wound-healing model, which represents a morphogenetic response to injury that is designed to restore physiological and anatomic function (Woodley et al. 1985). The biological processes involved in cutaneous wound healing include infiltration of inflammatory cells, fibroblast repopulation and new vessel formation, as well as keratinocyte migration and proliferation. Neutrophils are the first circulating inflammatory cells to be recruited to the site of the wound, presumably to decontaminate the wound from foreign debris and defend against possible infections. Clinical observations support the idea that neutrophils are important for efficient wound repair, as neutropenic individuals often have difficulty healing wounds (Nathan 2006). Impairment of leukocyte recruitment is also associated with delayed wound healing. Epithelialization and neovascularizaion following excisional wounds were decreased in C-X-C motif chemokine receptor 2 (CXCR2)-deficient mice (Devalaraja et al. 2000). Reduced neutrophil infiltration and delayed wound closure have also been reported in mice deficient in two formylpeptide receptors, Fpr1 and Fpr2 (Liu et al. 2014). It worth mentioning that, in these studies, blocking neutrophil infiltration did not affect the recruitment of other inflammatory cells such as monocytes, highlighting a direct role of neutrophils to wound healing. However, the specific effector functions of neutrophils that may contribute to wound healing remain unclear. Interestingly, neutrophil depletion resulted in delayed wound repair in aged mice but not in young mice, suggesting a functional change in neutrophils during aging (Nishio et al. 2008).

### Role of neutrophils in muscle injury and repair

There are many causes of muscle injury such as over-exercise or as a result of ischemia. Neutrophil infiltration has long been considered as the cause of excessive muscle injury (Pizza et al. 2005). Only recently have animal experiments suggested that neutrophils can also contribute to muscle growth and repair following injury. Mice treated by intraperitoneal injections of antisera to neutrophils and monocytes show a deficient regenerative response in a snake venom-induced myotoxicity model (Teixeira et al. 2003). Neutrophil and monocyte depletion also resulted in more tissue debris in the injured muscles in this model, suggesting that phagocytes removing tissue debris could contribute to the regenerative process (Teixeira et al. 2003). In another skeletal muscle stretch injury model, blocking CD11b and as a result neutrophil infiltration led to a markedly decreased initial regenerative response (Toumi et al. 2006). The authors thus hypothesized that reducing neutrophil infiltration results in not only reduced collateral damage but also a reduction in the repair response. Although the mechanism for this attenuated repair response is unknown, these intriguing studies suggested that neutrophil-mediated damage might be necessary for growth and repair. Further studies should focus on identifying the specific factors involved in each event, which could be targeted to manipulate selective events in an effort to achieve optimal healing.

Heart attacks or myocardial infarction are associated with a localized breakdown in the supply of oxygen to the organ, which results in the death of large numbers of cardiac muscle cells. A recent study found that neutrophils actively promote repair of the damage caused by heart attack (Horckmans et al. 2017). They do so by producing a factor called neutrophil gelatinase-associated lipocalin that mediates the differentiation of a distinct class of macrophages, which are the key players in the repair process. In another study, neutrophils and macrophages released the cytokine oncostatin M, which prompts a positive feedback loop in which oncostatin M galvanizes cardiomyocytes to produce regenerating islet-derived protein 3 β (REG3β) that in turn attracts additional macrophages to the damaged heart (Lorchner et al. 2015). As a rich source of interleukin-6 (IL-6), neutrophils may also directly contribute to cardiomyocyte proliferation via activating of the downstream effector of IL-6 receptor, the adapter protein signal transducer and activator of transcription 3 (STAT3), which controls satellite cell expansion and muscle repair (Han et al. 2015; Tierney et al. 2014). These studies highlight the pivotal roles of neutrophils as modulators of the healing response after myocardial infarction.

### Contribution of neutrophils to acute lung injury

Lung injury and repair includes many cell types and is relevant to the pathogenesis of most lung diseases. Acute lung injury can be induced by harmful stimuli such as pathogenic bacteria or inhalation of a toxin or particulate matter. The acute inflammatory response is characterized by accumulation of neutrophils in alveoli, increased pulmonary vasculature permeability and disruption of alveolar epithelium (Gonzalez-Lopez and Albaiceta 2012). Transmigration of neutrophils from the alveolar capillaries to the airspace causes damage to alveolar epithelial cells and is generally associated with a key alteration of alveolar function (e.g., plasma and interstitial fluid leakage into the airspace). However, there is some evidence that, under certain circumstances, neutrophil transmigration can occur without major barrier disruption. Furthermore, neutrophil accumulation also has a role in the repair and regeneration of the lung epithelium. This reparative function of the neutrophil accumulation is partially due to the clearance of epithelial debris from the sites of damage in order to create a clean matrix for regeneration of the epithelium (Hyde et al. 1999). In addition to this indirect contribution, neutrophils can also directly activate the repair response by activating lung epithelial cell proliferation. In mice treated with intratracheal LPS or keratinocyte chemokine, neutrophil transmigration activated the β-catenin signaling in alveolar type II epithelial cells, likely via elastase-mediated cleavage of E-cadherin (Zemans et al. 2011). Neutrophils also promote type II pneumocyte proliferation, which is essential for regenerating alveolar epithelium in a model of acid-induced acute lung injury. Proteomic analysis suggested that neutrophils promote multiple regenerative pathways, including MMP9, MMP2 and FGF1 (Paris et al. 2016).

### Neutrophils in central and peripheral nervous system injury

Neurons are normally unable to regenerate after injury to the central nervous system (CNS); however, this situation can be partially reversed by activating the innate immune system. Neutrophils are historically classified as unfavorable actors and have detrimental actions in the CNS (Gadani et al. 2015). However, there is now increasing evidence that neutrophils do not always cause more damage (Neirinckx et al. 2014). In one study using a sciatic nerve injury model, neutrophil depletion did not affect recovery of neurological function and peripheral axon regeneration (Nadeau et al. 2011). In another study, the authors used an optic nerve injury model and identified neutrophils as being the major contributor of oncomodulin, a neurotrophic factor that supports nerve regeneration following ocular injury. Antibody-mediated depletion of neutrophils blunted the zymosan-induced axonal regeneration (Kurimoto et al. 2013).

In one study, the authors sought to address the role of neutrophils in spinal cord injury by depleting neutrophils with antibody. These mice showed worse functional hindlimb recovery and delayed astrocyte reactivity, suggesting that neutrophils have a positive effect on the local glial response. However, as the authors used an antibody that can deplete both neutrophils and monocytes, it remains unclear whether neutrophils are indeed being beneficial. If so, the mechanism of neutrophils-mediated spinal cord injury repair response needs to be further studied (Stirling et al. 2009).

Beside the studies described above, human neutrophils have been shown to rapidly infiltrate the hematoma associated with bone fractures and synthesize fibronectin^+^ extracellular matrix before stromal cells infiltrate and synthesize bone tissue, thus contributing to bone regeneration (Bastian et al. 2016). Additionally, neutrophil-borne cathelicidin (LL-37 in human) promoted re-endothelization and thereby limiting neointima formation and contributed to arterial healing after injury (Soehnlein et al. 2011). Other than releasing factors that promote tissue repair, activated neutrophils can also generate microvesicles that contain nucleic acids and proteins, which have an overall anti-inflammatory and pro-resolving effect on myeloid cells (Dalli et al. 2013; Gasser and Schifferli 2004). Neutrophils also accelerate the inflammatory resolution through localized oxygen depletion in acute intestinal inflammation and neutrophil depletion aggravates the mucosal damage (Campbell et al. 2014). These data suggest that neutrophils have a variety of important biological functions far beyond cytotoxicity and further research is needed to uncover the mechanisms that regulate these distinct functions.

### Fate of neutrophils and the subsequent influence on tissue repair: neutrophil reverse migration.

Neutrophils are short-lived cells under steady condition but, once migrated into tissue, they are exposed to survival signals to increase their life-span. It has long been accepted that, in a successful acute inflammatory response that completely resolves, neutrophils are cleared from inflamed tissue in a timely fashion (Soehnlein and Lindbom 2010). When this clearance does not occur appropriately, neutrophils undergo necrosis and release intracellular contents that can damage the tissue and extend the inflammatory phase. However, neutrophils do not always die at the site where they were recruited. Early evidence suggested that neutrophils accumulating at inflamed sites do not undergo apoptosis followed by phagocytosis by macrophages. In a rat model of glomerular capillary injury, the authors infused radiolabelled neutrophils to track the fate of these cells and found that the majority of neutrophils that entered inflamed glomerular capillaries were able to return to the main circulation instead of becoming apoptotic at the site of inflammation (Hughes et al. 1997). More recent studies have shown that neutrophils can leave the site of tissue damage in a process termed neutrophil reverse migration, which means that interstitial tissue-infiltrated neutrophils migrate away from inflamed sites. Several studies directly visualized this event in vivo in zebrafish larvae following tissue injury and have suggested that reverse migration is a possible mechanism to locally resolve inflammation and repair injury (Mathias et al. 2006; Powell et al. 2017; Robertson et al. 2014). A recent study combining intravital imaging and photoactivation techniques also demonstrated that murine neutrophils perform reverse migration from an injury site, moving back to circulation and eventually home back to the bone marrow (Wang et al. 2017). However, neutrophils have also been reported to re-enter the vasculature in a distinct process referred to as neutrophil reverse transendothelial migration (rTEM) in ischemia/reperfusion injury. In this model, activated neutrophils migrated from the abluminal side to the luminal side of the blood vessel and were redistributed to other locations in the body, contributing to second-organ tissue injury (Colom et al. 2015; Woodfin et al. 2011). This observation is also supported by clinical evidence that human patients with acute pancreatitis who developed acute lung injury were found to have a higher level of neutrophils carrying rTEM markers in their circulation (Wu et al. 2016). However, these studies left open the question regarding how reverse transendothelial migration would affect the initial inflammatory response. Much more work needs to be carried out to further clarify the phenotype and the fate of reverse-migrated neutrophils and the implications in human disease.

## Concluding remarks

With the development of advanced technologies such as intravital imaging, the past decade has clearly been a golden age for neutrophil biology research. Neutrophils have traditionally been considered to cause collateral tissue damage; however, recent studies indicate a clear protective role for neutrophils during resolution and repair. Although there may well be a threshold value at which the positive impact of the neutrophils is adversely affected, from a therapeutic perspective, many of the studies described above provide potential therapeutic targets and approaches other than simply depletion of the neutrophils for the treatment of inflammatory disorders.
